# Light Rare Earth Elements in Plants: Environmental Sources, Bioavailability, Uptake, Nutrient Interactions, Mechanisms, and Concentration-Dependent Physiological and Signaling Reactions

**DOI:** 10.3390/plants15192947

**Published:** 2026-09-27

**Authors:** Saurabh Pandey, Debanjana Saha, Pratibha Machagondanahalli Dharmappa, Prajjal Dey, Jyoti Chauhan, Rintu Jha, Prince Choyal, Udit Nandan Mishra, Sunita Gupta, Narendra Kumar Gupta, Mahendra Singh, Shahid Ahmed, Rajesh Kumar Singhal, Radha Sivarajan Sajeevan

**Affiliations:** 1 Department of Molecular Biology and Biotechnology, Indira Gandhi Agricultural University, Raipur 492012, India; 2 Central Muga Eri Research and Training Institute (CMER&TI), Lah Jorhat 785700, India; 3 ICAR-Indian Institute of Horticultural Research, Bengaluru 560089, India; 4 Division of Basic Science & Humanities, Faculty of Agriculture, Sher-e-Kashmir University of Agricultural Sciences and Technology, Jammu 190017, India; 5 ICAR-Indian Grassland and Fodder Research Institute, Jhansi 284003, India; 6 ICAR-Indian Institute of Soybean Research, Indore 452001, India; 7 Faculty of Agriculture, Sri-Sri University, Cuttack 754006, India; 8 Department of Plant Physiology, Sri Karan Narendra Agriculture University, Jobner 303329, India; 9 Department of Plant Protection Biology, Swedish University of Agricultural Sciences, 23422 Lomma, Sweden

**Keywords:** LREEs, abiotic stress, bioavailability, growth and development, mineral nutrition, plant signaling, photosynthesis

## Abstract

Rare earth elements (REEs) are traditionally regarded as emerging environmental contaminants. Due to their increasing anthropogenic release and interactions with essential mineral nutrients, they are attracting growing interest for their potential applications in sustainable agriculture. The REEs are a unique group of 17 chemically similar elements, including scandium (Sc), yttrium (Y), and lanthanides. Most existing reviews treat REEs as a single group, with limited emphasis on concentration-dependent uptake mechanisms or species-related responses. However, based on atomic weight, REEs are classified into light and heavy rare earth elements (LREEs/HREEs). LREEs enter the plant system through multiple routes-fertilizer, irrigation, and mining residues; however, their cellular roles and modes of action remain insufficiently understood. Accumulating evidence indicates that LREEs exert concentration-dependent effects on plant growth and metabolism, including decreased growth, oxidative stress, modulation of photosynthesis, chlorophyll and carotenoid accumulation, antioxidant metabolism, secondary metabolite production, and enzyme activities. LREE uptake occurs predominantly via calcium-permeable channels, with limited involvement of transporters, followed by root sequestration and restricted long-distance translocation. At the cellular level, LREEs influence redox balance, membrane stability, chloroplast ultrastructure, and mineral nutrient homeostasis, and interact with multiple signaling pathways, including calcium- and hormone-mediated networks. Most studies on LREEs focused on lanthanum and cerium, attributed to their abundance in soil. Unlike previous reviews addressing REEs broadly, this review specifically focuses on LREE-mediated regulation of plant growth and development, mineral nutrition with emphasis on concentration-dependent responses, uptake and transport, and associated signaling mechanisms and hormonal interactions. Key gaps in current knowledge regarding LREE molecular targets, transport, dose-dependent mechanisms, molecular regulation, and species-specific responses are highlighted to guide future research. By integrating environmental entry routes with cellular and molecular responses, this review provides a mechanistic framework for future studies on LREE plant interactions.

## 1. Introduction

Rare earth elements (REEs) are f-block trace elements that have been shown to be beneficial for various plant processes [1,2,3]. According to the International Union of Pure and Applied Chemistry (IUPAC), REEs comprise 15 elements from the lanthanide (Ln) series, with atomic numbers ranging from 57 to 71 [4,5]. These elements are known as “rare elements” since they are distributed globally in soils but lack large deposits and ores in pure form [6,7]. Based on atomic number, the REEs are divided into two groups: light rare earth elements (LREE) and heavy rare earth elements (HREE). LREE includes elements ranging from atomic number 57 to 63, i.e., lanthanum (La), cerium (Ce), praseodymium (Pr), neodymium (Nd), promethium (Pm), samarium (Sm), and europium (Eu). In contrast, HREEs include elements ranging from atomic number 64 to 71, i.e., gadolinium (Gd), terbium (Tb), dysprosium (Dy), holmium (Ho), erbium (Er), thulium (Tm), ytterbium (Yb), and lutetium (Lu) [4]. Although REEs comprise both LREEs and HREEs, this review focuses specifically on LREEs because their occurrence, plant uptake, and reported effects on growth and development have received greater attention than those of HREEs. LREEs are more critical in their abundance in the soil based on the Oddo-Harkins rule. LREEs such as La, Ce, Pr, and Nd also provide a useful framework for examining concentration-dependent plant responses, including growth stimulation at low concentrations and toxicity at higher concentrations. Focusing on LREEs therefore allows a more mechanistic assessment of their uptake, transport, signaling, and developmental effects, while identifying research gaps relevant to crop production and future applications. Because most published physiological studies have focused on La and Ce (due to their abundance), this review primarily discusses LREEs, while the term REEs is used only when referring to the entire rare earth element group. Based on this theory, elements with even atomic numbers are more abundant in nature than their nearby elements with odd atomic numbers because of the greater relative stability displayed by atomic nuclei [8,9,10]. Due to their widespread use in contemporary agriculture, industry, and green technologies, REEs, especially light rare earth elements (LREEs; e.g., La, Ce, Pr, Nd), have drawn more attention as new environmental pollutants. Their distribution and bioavailability in soils have been greatly affected by their accelerated release into terrestrial ecosystems through mining operations, fertilizers, wastewater irrigation, and atmospheric deposition [11,12]. Complex soil physicochemical characteristics, including pH, organic matter, and mineral composition, govern the bioavailability of REEs and ultimately regulate plant uptake [13,14]. However, these elements interact with plant essential nutrients to increase their availability [15,16]. Due to their similar ionic properties, plants mostly absorb LREEs through apoplastic and symplastic channels in their root systems, frequently using transport systems similar to those of critical ions like Ca^2+^ [17,18]. In addition, recent research demonstrates that foliar uptake from airborne particles can increase the channels of entry for REE buildup in plants [19,20].

Rare earth elements are considered an essential component of natural earth systems. Long-term weathering history, texture, pedogenic processes, anthropogenic activities, and organic matter content convincingly demonstrate the abundance of these soils. REEs are found with silicates, PO_4_^3−^, oxides, halogens, and CO_3_^2−^ compounds [9,21,22]. Distribution of the LREEs in the Earth’s crust is more common than the HREEs, and their ratio changes with geological events. REEs are usually present in Earth’s crust, with lower subsurface concentrations ranging from 54 to 130 mg kg^−1^. According to Redling [23,24], REEs are abundant in the soil in the order of Ce≥, La≥, Sc, Nd, Sm, Pr, Gd, Dy, Eu, Tb, Er, Yb, Ho, Lu, and Tm.

The significance of REEs in influencing plant processes was first reported in 1917 [25,26]. They observed that Ce-mediated elements enhanced water flow and plant physiological activity. Later in the twentieth century, the application of REEs in agriculture was reported to stimulate growth [27,28]. On average, a 5–15% increase in yield has been reported with the application of REEs. Besides acting as a growth stimulant, REEs are key regulators that protect plants against biotic and abiotic stresses [2,23]. These elements can have a positive or negative impact on plant processes depending on their concentration and the timing of application. In general, low concentrations of REEs appear to be beneficial for plant growth, whereas higher concentrations can be toxic, leading to oxidative stress, cell membrane damage, and disrupted metabolic processes. Moreover, it has been observed that the regular use of REEs may lead to adverse effects due to their overaccumulation in soil, water, and the atmosphere [29]. Likely, the REEs transcriptionally modulate metal ion transporters, providing plants with access to essential minerals. This dual role underscores the importance of understanding optimal REE dosages and application methods for agricultural use. Furthermore, environmental contamination and bioaccumulation concerns highlight the need to carefully assess the long-term impacts of REE use in farming practices [30]. Consequently, it is imperative to decipher the mechanism underlying REEs effect on plant physiological processes to ensure global food security.

The physiological effects of REEs in plants include increased leaf area, higher chlorophyll and carotenoid contents, enhanced activity of photosynthetic enzymes, higher accumulation of endogenous phytohormones, and strengthened antioxidant defense [31,32,33]. However, their precise functional mechanisms and the crosstalk with phytohormonal signaling remain poorly understood. Additionally, the mechanism of action of REE minerals within plants remains controversial, with different hypotheses proposing their localization in the apoplast or within cellular organelles such as the vacuole, nucleus, and chloroplast. The diverse roles of different REEs in plant growth and development are highlighted and illustrated in Figure 1. Therefore, a comprehensive understanding of their biogeochemical cycle and transport pathways is essential to elucidate their mechanisms of action in plants.

Exposure to REEs in crops is predominantly from LREEs, due to their presence in phosphate fertilizers, industrial byproducts, and mining residues. Although there are increasing reports on the growth-stimulating effects of LREEs, less information is available on their mechanisms, uptake, and concentration thresholds. Therefore, this review summarizes and discusses the recent advances in LREE uptake, transport, and their physiological roles, particularly in key processes such as seed germination, nutrient uptake, photosynthesis, antioxidant defense, and signaling networks. It also addresses their emerging function in plant stress tolerance and developmental regulation, aiming to guide future research for sustainable crop production.

## 2. LREE Sources and Global Distribution

Light rare earth elements are widely dispersed throughout the Earth’s crust and are becoming more mobilized as a result of human activities like mining, industrial discharge, fertilizer application, and recycling electronic trash. Recent findings have demonstrated that LREE concentrations are higher in mining-intensive areas and agricultural soils that receive phosphate fertilizers, which causes them to build up in the soil–plant system [34,35]. LREEs are growing contaminants of concern in agroecosystems due to atmospheric deposition and wastewater irrigation, which further contribute to their global dispersion.

According to the United States Geological Survey’s 2020 data, global REE reserves are estimated at around 120 million tonnes. The REE deposits have been reported in 34 countries. With around 36.7% of the deposits, China leads, followed by Brazil and Vietnam with 18.3% shares. Russia, India, and Australia possess significant REE deposits, contributing about 10%, 5.8%, and 2.8% to global reserves, respectively. Nevertheless, the extraction of REEs from economically viable mines has a limited scope compared to other, more diverse rare-earth mines [36]. The amount of LREE in the original soil varies between 40 and 900 mg kg^−1^ in different parts of the world [37]. The soil near tailings sites generally exhibits high concentrations of LREEs, resulting in unfavorable soil structure, poor organic matter content, and impaired microbial and nutritional compositions [38]. Therefore, the effect of LREE contamination is more pronounced in ecosystems near tailings sites. Another study demonstrated that the range of LREE was considerably higher than that of HREE, with La, Ce, Pr, Nd, and Sm showing dominance near mining areas. Among them, La and Ce accounted for 9.2–24.6% and 13.8–48.2% of ΣREEs, respectively [39]. Once exogenous LREEs enter the soil, more than 99.5% of them are absorbed by the solid phase, and only a tiny amount becomes dissolved in the water available in the soil.

The LREEs can be found in oxides, silicates, CO_3_^2−^, PO_4_^3−^, and halogen compounds in nature. The most common and diverse deposits of LREEs exist in the form of CO_3_^2−^ and fluorocarbonates, oxides, silicates, and PO_4_^3−^ [9,21]. The primary sources of LREE in agricultural fields encompass different geological formations such as carbonatites, magmatic magnetite-hematite bodies, iron oxide–copper–gold (IOCG) deposits, peralkaline igneous systems, xenotime–monazite accumulations in mafic gneiss, ion-absorption clay deposits, and placer deposits containing monazite-xenotime minerals [40]. LREEs are mainly found in carbonatites and igneous rocks derived from carbonate-rich magmas [41]. The LREEs are produced from six primary mineral sources: bastnaesite [(Ce, La) (CO_3_) F], monazite [(Ce, La) PO_4_)], xenotime (YPO_4_), loparite [(Ce, Na, Ca) (Ti, Nb) O_3_], apatite [(Ca, REE, Sr, Na, K)_3_Ca_2_(PO_4_)_3_(F, OH)], and ion-adsorption clays [42]. Among these, bastnaesite is considered the chief source of LREE minerals. The natural sources and concentration of LREEs are highlighted in Table 1.

Besides natural LREE sources, mining and smelting emissions, and anthropogenic sources also result in soil enrichment with LREEs [48]. The other LREE sources include permanent magnets/voice coil motors, fluorescent lamp phosphorus, Fe-Nd-B magnets, Sm-Co magnets, TV tubes, computer monitors, coal and coal by-products, iron ore tailings, apatite, phosphate by-products, mine waste and/or industrial landfill by-products (red mud, phosphogypsum waste, coal ash, RE metallurgical slags, incinerator ash, and wastewater streams) (Table 2). Kumar et al. [49] found that the Brahmaputra River in India is a rich source of LREEs. Moreover, REE reservoirs in landfills contain disposed electronic and electrical waste [50]. According to Costa et al. [30], soil sediments collected from nearby fertilizer industries in Southern Brazil showed high contamination with LREEs. Secondary sources of LREEs include the reclamation of waste, electronic waste such as computers and laptops at the end of life, and NdFeB magnets, which contain considerably higher amounts of recyclable Nd [51]. Similarly, Nd, La, Ce, and Pr are recycled from the end-of-life Ni-MH batteries used in vehicles [52]. Omodara et al. [51] described secondary sources of LREE resulting from recycling. The abundance of REEs in primary and secondary sources in the agricultural field underscores the need to examine their physiological effects on crop growth and productivity.

In agricultural soils, LREE bioavailability depends on factors such as soil pH, redox conditions, and organic matter content. For example, acidic soil increases the solubility of trivalent cations, which in turn exposes the plants to them for uptake. Whereas organic matter forms complexes, leading to LREE immobilization. Exposure of crops to LREEs often occurs due to anthropogenic activities, including phosphate fertilizer application, mining tailings, and industrial waste streams.

## 3. LREEs Uptake and Transport

LREEs are mostly taken up by roots via apoplastic and symplastic pathways; because of their similar ionic radii and charge properties, they frequently resemble calcium ions (Ca^2+^). According to Wang et al. [17], certain transport mechanisms, such as ion channels and carrier proteins, enable their entry and translocation throughout plants. Similarly, root exudates, especially organic acids, are essential for improving LREE solubility and bioavailability in the rhizosphere, which in turn affects their absorption efficiency.

Deciphering the physiological functions of mineral elements requires in-depth knowledge of their uptake and transport mechanisms. The uptake mechanism of different LREEs includes the valency of an element, Casparian strips, low-molecular-weight organic acid complexes, plant root exudates, organic and inorganic ligands, and chelating agents. Primarily, the plasma membrane (PM) affinity affects the absorption of an element [64,65]. Secondly, the adsorption of LREEs is influenced by several physicochemical soil properties, including redox potential, pH, and cation exchange capacity (CEC). Numerous studies have assessed the relevance of LREEs and their complexation with diverse ligands under varied pH conditions. For instance, neutral pH (7 ≤ pH ≤ 9) favors LREE complexes with CO_3_^2−^, [which includes LnHCO_3_^2+^, LnCO_3_^+^, and Ln (CO_3_)^2−^], and with phosphate, [LnH_2_PO_4_^2+^, LnHPO_4_^+^, Ln (HPO_4_)^2−^, and LnPO_4_] [66]. LREE adsorption capacities are also closely correlated with soil CEC. Generally, lower pH levels are associated with enhanced CEC, redox potential, mobility, and consequent availability of LREE cations.

Considering soil organic matter, it is another important control of LREE retention and mobility. Organic functional groups of humic substances can bind REE^3+^ ions by adsorption and complexation and thus affect their partitioning between solid and solution phases. Depending on the pH of the soil and the type of organic matter, these interactions can either increase retention by surface complexation or increase mobility by the formation of soluble organic complexes [12,67]. Similarly, CEC also affects REE retention because negatively charged sites associated with clay minerals and soil organic matter are important exchange and adsorption sites for the trivalent REE cations. Recent studies have suggested that CEC, in conjunction with clay mineralogy, soil pH, and oxide content, may influence REE adsorption and mobility in soils [68]. The redox potential further modulates REE behavior by changing the stability and dissolution of Fe- and Mn-bearing phases that constitute key sorption sites. Changes in redox conditions of soils can thus facilitate REE release, redistribution, and fractionation [10,67]. To summarize, LREE bioavailability should be considered as an interplay of effects of pH, organic matter, CEC, redox conditions, mineralogy, and ligand availability instead of a response to any single soil property [12,67,68].

## 4. Root Surface Interactions and Entry Pathways

Calcium-permeable channels are the predominant root uptake pathway of LREEs in ionic forms due to their similar ionic radii. Different studies suggest that LREEs interact competitively with calcium-binding sites during transport. Moreover, there is no dedicated high-affinity transporter system for LREEs. A few reports also suggest nanoparticle-mediated LREE uptake. Unlike ionic LREEs, nanoparticles may undergo particle-associated uptake, including endocytosis, or physicochemical transformation and partial dissolution at the root surface or in the rhizosphere, releasing ionic species that can subsequently enter through conventional ion-uptake pathways [69]. Root exudates, such as low-molecular-weight organic acids (OAs), are essential for metal chelation or complex formation, thereby affecting soil metal solubility and plant uptake. Further, OAs are also involved in metal complexes in hyperaccumulator shoots, which exist in sufficient amounts in most terrestrial plants. At cytosolic pH 7.5, histidine has the maximum association constant for the formation of a complex of OAs and proteinaceous amino acids [65]. Consequently, the release of carboxylate also explains the hyperaccumulation of REE in Proteaceae species [70]. A recent review reported that REE exposure significantly modifies root system architecture, elongation, and exudation patterns across diverse crop species, underscoring root physiological plasticity that supports such metal-complexation and hyperaccumulation processes [71]. Examples of some hyperaccumulator plants and their uptake mechanisms are highlighted in Table 3.

Collaborative uptake pathways are also common for LREE transport in plants. Similar ionic radii facilitate this type of uptake; for instance, the trivalent LREEs and divalent calcium (Ca^2+^) show many similar physicochemical characteristics in plants and other biological systems. It has been reported that La^3+^ inhibited intracellular and extracellular Ca^2+^ and replaced Ca^2+^ at extracellular binding sites. Furthermore, La competes with calcium for calcium-binding proteins, e.g., calmodulin (CaM), although the binding affinities differ across experimental setups [90]. Thus, Ca^2+^ channels at the PM of root cells are recognized as specific channels for La uptake [91,92]. Similarly, La has been shown to competitively bind to Al^3+^ binding sites on the PM, potentially mitigating Al toxicity [93]. Yuan et al. [75] hypothesized that LREEs, including La, may utilize existing Al^3+^ and Ca^2+^ uptake pathways to enter the root cells. However, to date, only one plant REE-specific transporter has been partially studied. NREET1, identified in *Dicranopteris linearis*, is a plasma membrane-localized transporter that belongs to the natural resistance-associated macrophage protein (NRAMP) family. However, it is not involved in Al^3+^ or Ca^2+^ transport [94]. An important knowledge gap is whether functional homologs of NREET1 occur in conventional crop plants. NREET1 has been characterized in a rare earth element hyperaccumulator fern, but its conservation, expression, and functional contribution to LREE uptake and transport in major crops remain largely unresolved. Comparative genomics combined with gene expression, transporter assays, and CRISPR-based functional validation will be required to determine whether NREET1-like proteins represent a conserved mechanism for LREE transport or a specialized adaptation of hyperaccumulator species.

In contrast to ionic LREEs, LREE-containing nanoparticles may follow distinct entry pathways and can also undergo physicochemical transformation before or during plant uptake. Endocytosis has been proposed as one mechanism for nanoparticle-associated LREE entry into plant cells [69]. Cheng et al. [95] demonstrated that foliar application of La^3+^ can trigger systemic endocytosis, allowing its entry into epidermal cells of leaves. This process was found to require the coordinated action of AtrbohD (an NADPH oxidase), which mediated ROS generation and jasmonic acid (JA) production. Root exudates, such as succinic acid, can chemically transform cerium dioxide (CeO_2_) nanoparticles into Ce^3+^ ions, facilitating their absorption by root cells [96]. Application of CeO_2_ nanoparticles (NPs) in radish revealed that the uptake of Ce occurs through a cocktail of CeO_2_, Ce ions (Ce^3+^/Ce^4+^), and Ce complexes in root cells. However, Ce particles larger than 20 nm, which exceed the size exclusion limit of root cell walls, are generally unable to pass through intact roots, although they can enter through damaged root tissues or via foliar routes [97]. Thus, ionic LREEs and LREE-containing nanoparticles should be considered distinct but interconnected exposure forms: ionic LREEs primarily interact with ion channels and transporters, whereas nanoparticles may undergo direct particle-associated uptake or transformation into ionic species before entering conventional uptake pathways. The main interface for LREE uptake is the root surface, where their bioavailability is determined by interactions with soil particles, root exudates, and microbial populations. Before entering through ion channels or transporters, LREEs can attach to mucilage and root cell walls. Roots emit organic acids that make LREEs more soluble and easier to absorb. Recent research highlights the significance of root exudation and rhizosphere chemistry in controlling LREE penetration into plant systems [13,66].

## 5. Radial Transport and Xylem Translocation

Following entry, LREEs exhibit limited xylem mobility and preferential accumulation in root tissues. Xylem loading appears closely linked to transpiration rate and aquaporin activity rather than active long-distance transport mechanisms. In xylem sap and shoot tissues, Ce is found primarily as a mixture of Ce^3+^ and Ce^4+^, whereas in the phloem, CeO_2_ was dominant [98]. Moreover, Ma [99] demonstrated that cycloheximide pretreatment affects protein synthesis, significantly alters water transpiration, and alters aquaporin activity in soybean. As a result, cerium (Ce) content in root tissues decreased by 67%, indicating that xylem-mediated transport and aquaporin channels play a crucial role in Ce uptake and movement within the plant. Following root uptake, apoplastic and symplastic routes carry LREEs radially across root tissues before they enter the xylem for long-distance translocation. Their entry into the circulatory system is controlled by the Casparian strip, which functions as a selective barrier. Recent studies showed that complexation with organic acids affects LREE mobility in xylem and increases their translocation to aerial plant parts [17,66]. Despite the increasing usage of REEs in modern technology, little is known about how they affect aquatic ecosystems, even though they are now recognized as an emerging pollutant. The effects of REEs (e.g., Ce, Nd, Gd, Dy, Yb, Ho, and Lu) in chloride form have been demonstrated on aquatic biomonitoring plant species, *Lemna minor* L. [100].

## 6. Subcellular Sequestration

At elevated exposure levels, LREEs accumulate in cell walls and vacuoles, indicating sequestration driven by detoxification rather than functional signaling. The microscopic studies reveal that LREEs can infiltrate plant tissues via the apoplast of root meristems, from which they migrate into the stele of roots and shoots. This phenomenon has been observed in several plant species, including *Hordeum vulgare* L., *Salicornia virginica* L., and *Spartina alterniflora* [101]. Under prolonged exposure to high concentrations of La^3+^, its accumulation has also been detected in the cytosol of root cells; however, this is typically associated with toxicity-related responses rather than normal physiological uptake. Notably, hyperaccumulator species possess the ability to solubilize LREEs from the soil and compartmentalize them into specific vacuolar structures. This improves the efficacy of LREE uptake by the roots, translocation to leaves, and sequestration within plant shoot cells. Despite these findings, the uptake and transport mechanisms of many other LREEs remain poorly characterized, controversial, and underexplored. To reduce toxicity, LREEs are segregated within plant cells after absorption. Plant organelles such as vacuoles, cell walls, and chloroplasts are the main locations for subcellular sequestration. On the other hand, LREE detoxification and storage are made easier by binding with organic ligands, such as proteins and organic acids. However, vacuolar sequestration is found to be a key mechanism for tolerance, especially in hyperaccumulator species [17,72]. The uptake and transport mechanisms of LREEs are highlighted in Figure 2.

## 7. Crosstalk of LREEs with Essential Mineral Elements

Mineral elements are vital for optimal plant growth and development. Interactions between LREEs and essential nutrients depend on external concentrations, soil chemistry, and transporter competition rather than on uniform synergistic or antagonistic behavior. The deficiency or excessive concentrations of these elements can lead to reduced physiological plant growth or even mortality [102]. Several studies indicate that LREE ions regulate plant growth by modulating the concentrations of essential nutrients [27]. The availability of these nutrients is influenced by mechanisms of antagonistic or synergistic interactions, as chemically similar elements compete for binding sites and absorption into plants [103]. Soil conditions also affect the relationships between REE and other elements. Hu et al. [27] reported that increased N and K and high PO_4_^3−^ fertilization enhanced the uptake of LREEs. Similarly, Cao et al. [104] reported a positive correlation between the release of Fe and Mn and LREE absorption. In *Saxifraga paniculata*, Fe and Al levels were positively associated with Y and Ce uptake, indicating analogous behavior in soil and plant uptake mechanisms [105]. In addition, LREEs were found to interact both antagonistically and synergistically with mineral nutrients. Plant species, soil conditions, and concentration all had a significant impact on the responses. While high concentrations of LREEs may impede nutrient uptake and disrupt ionic homeostasis, low to moderate concentrations may improve nutrient and physiological process uptake. According to recent research, therapies based on La and Ce can change the nutrient status of many crops. Instead of a consistent nutritional effect, responses exhibit a distinct hormetic pattern that is dependent on dose and soil-moisture conditions [12]. The interaction of LREEs with Ca-mediated signaling, membrane binding sites, and rhizosphere ligands—whose availability is similarly controlled by soil pH, organic matter, and mineral interactions—may be responsible for these disparate reactions [11]. Therefore, at optimal LREE levels, a potential synergistic impact would result from improved root activity or nutrient-use efficiency, but antagonistic effects at higher doses might result from competition for uptake or binding sites and disruption of nutrient homeostasis. Therefore, rather than being consistently positive or negative impacts, LREE-nutrient interactions should be regarded as dose-, species-, and soil-context-dependent associations.

LREEs, when present in optimal concentrations, enhance ion transport across plant cell membranes by stimulating membrane-bound proteases, thereby increasing the accumulation of mineral elements. For example, cerium supply between 1 and 5 µM enhances zinc accumulation through shared ZIP transport pathways, while concurrently reducing iron uptake through competitive inhibition of iron-regulated transporters under acidic conditions. However, negatively charged compounds on the plasma membrane can interact with REE ions, disrupting typical ion gradients and impeding mineral uptake [106]. Although limited information exists on how the timing of LREE application influences mineral distribution, Brioschi et al. [64] observed a correlation between LREE concentrations in tree roots and Fe-Mn oxyhydroxides in limestone soils, as well as Fe-oxyhydroxides in clay-humus soils. Iron shares similar absorption mechanisms with LREEs. Application of 25 and 100 µM Ce in rice increased shoot zinc content by 69.6% and 47.8%, respectively, but reduced root iron by 25.9% [107]. In soybeans, the presence of cadmium (Cd) increased Ce uptake, although CeNPs did not affect Cd levels. Ce also formed a root barrier to Cd [108]. Nevertheless, 1000 mg/kg of CeNPs reduced Ca^2+^ and molybdenum levels while increasing phosphorus and copper levels in soybean pods, potentially affecting pod quality [109]. In *Vigna angularis*, LaNO_3_ treatment improved phosphorus use efficiency and mitigated deficiencies affecting photosynthesis and chlorophyll [110]. Similarly, in *Boehmeria nivea*, REE exposure (80–800 µmol/L) increased phosphorus but decreased Ca, Mn, K, Mg, Cu, and Zn [111]. Potassium (K) content decreased at lower La concentrations (1 and 2.5 µM) but remained stable at 5 and 10 µM, with a 2.5 µM dose increasing K in root cells. Magnesium and Ca^2+^ concentrations also differ from the La concentration in root and shoot tissues [112].

La(NO_3_)_3_ has been shown to enhance root architecture, antioxidant defense, photosynthesis, and water uptake in adzuki beans under phosphorus deficiency [110]. In *Brassica juncea*, reduced Fe, Mn, and Zn were observed in both roots and shoots under hydroponic conditions [113]. Moreover, La concentrations ≥ 1 mg/L restricted Cd translocation to shoots, while high Cd levels reduced La accumulation in shoots but not in roots. This antagonistic interaction reduced uptake of both elements by up to 35%, associated with downregulation of transporter proteins NRAMP5 and HMA2 [114]. Additionally, Cd toxicity disrupted La-Cd and La-Ca interactions through calmodulin-mediated suppression of Nramp5 expression in wheat [114]. Pr, another LREE, mitigated Cd toxicity and enhanced root growth in rice [115] and improved antioxidant enzyme activity in maize [116]. Neodymium chloride (NdCl_3_) decreased chromium uptake by 32.2% in roots and 33.7% in leaves, thereby alleviating Cr toxicity in wheat [117]. Furthermore, magnetic Nd treatment significantly improved coriander seed germination, with a 98.3% success rate and a shortened germination period of 8.1 days compared to 11.8 days in the control group [118]. Though data on other LREE interactions remain limited, existing evidence suggests they are likely transported via nutrient-specific transporters, influencing uptake and nutrient dynamics in plants. A recent study reveals a complicated relationship between LREEs and plant growth regulators such as abscisic acid, auxins, and cytokinins. Plant developmental processes and stress adaptation mechanisms can both be modified by LREEs through their effects on hormone production, transport, and signaling pathways [76]. Since LREEs can either promote or decrease hormone-mediated responses depending on their concentration and environmental context, this crosstalk is especially significant under abiotic stress settings [19].

## 8. Impact of LREEs on Critical Plant Physiological Processes

Physiological responses to LREEs, including La, Ce, Nd, and Pr, exhibit concentration-dependent patterns. Low external supply between 0.5 and 2 µM frequently increases chlorophyll content, net photosynthetic rate, and biomass accumulation by 10 to 25 percent in cereal crops. In contrast, concentrations exceeding 50 µM disrupt chloroplast ultrastructure, reduce photochemical efficiency, and suppress growth. These beneficial effects are attributed to the LREEs’ ability to mimic or replace essential ions like calcium (Ca^2+^) and magnesium (Mg^2+^) in critical biochemical pathways, including those regulating Rubisco activity and electron transport in photosynthesis.

Plant physiology is greatly affected by LREEs in a concentration-dependent manner. They can improve plant growth and stress tolerance at low concentrations by promoting photosynthesis, enzyme activity, and seed germination. On the other hand, excessive buildup causes oxidative stress, compromises membrane integrity, and interferes with the absorption of nutrients [72,76]. These two outcomes demonstrate how hormetic LREEs act in plant systems. The concentration-dependent effects of LREEs on the crucial plant physiological and biochemical processes were highlighted in Table 4. However, when LREE concentrations exceed optimal thresholds, they become toxic. This toxicity can impair cellular metabolism, damage organelles such as chloroplasts, and reduce overall plant vitality. Plants often respond to high LREE levels by compartmentalizing excess ions, especially in root tissues, which tend to accumulate more LREEs than leaves [119]. This compartmentalization mechanism helps mitigate toxicity but may limit the beneficial physiological effects observed at lower concentrations. Furthermore, LREE effects vary depending on plant species, developmental stage, environmental conditions, and the chemical form of the applied REEs [120]. Thus, optimizing LREE concentration is crucial for maximizing their beneficial effects while minimizing phytotoxicity. The dual nature of LREEs is particularly evident in their role in photosynthesis: low concentrations enhance chlorophyll content, CO_2_ assimilation, and Rubisco activity, whereas high levels disrupt chloroplast structure and reduce photosynthetic rate. These contrasting effects are depicted and summarized in Table 4.

## 9. Interaction of LREEs with Signaling Molecules

### 9.1. LREEs and Calcium Signaling

Calcium (Ca^2+^) is a vital plant nutrient that acts as a secondary messenger in numerous cellular processes, including cell wall formation and stress response regulation [137]. LREEs exert dual effects on calcium signaling depending on exposure level. At low concentrations, transient interference with membrane potential may trigger short-lived cytosolic calcium signals. At higher concentrations, lanthanum and cerium block calcium-permeable channels, suppress calmodulin-mediated signaling, and impair downstream kinase activation. Recent research has demonstrated that LREEs have a major impact on plant signaling networks by modifying the pathways for nitric oxide (NO), calcium signaling, and reactive oxygen species (ROS). Changes in intracellular Ca^2+^ levels brought on by LREEs can set off downstream signaling cascades that impact stress responses and gene expression [76]. Moreover, LREEs can interfere with ROS homeostasis by acting as stress inducers at higher concentrations or as signaling modulators at lower levels [72]. LREEs cause concentration-dependent physiological reactions in plants once they are absorbed. As a result of their hormetic activities, they can boost photosynthetic efficiency, increase stress tolerance, and promote plant growth at low concentrations [121]. Higher concentrations of REEs, however, cause phytotoxicity by producing ROS, damaging membrane integrity, and interfering with nutritional homeostasis, especially through competing with Ca^2+^ [71]. Additionally, these components influence plant adaptability and stress responses by modulating important signaling pathways such as calcium signaling, ROS-mediated responses, and hormone control [76]. Additionally, current developments emphasize the significance of soil–plant continuum processes, rhizosphere interactions, and root exudates in regulating REE mobility, accumulation, and physiological effects in plants [80]. In general, assessing LREEs’ dual role as both beneficial and toxic elements in plant systems, especially under changing environmental conditions, requires an understanding of their environmental sources, bioavailability, uptake mechanisms, and concentration-dependent physiological and signaling responses.

LREEs, such as La^3+^, can mimic or compete with Ca^2+^, thereby disrupting calcium-dependent physiological mechanisms. LREEs can enter cells through calcium-permeable channels in the PM and interact with specific REE-binding proteins, thereby influencing several physiological pathways, including chlorophyll activity, phytochrome red levels, proline accumulation, and regulation of ROS. These interactions help maintain PM stability and enhance plant tolerance to stress [138]. However, excess Ln^3+^ ions can outcompete Ca^2+^ for binding sites on cellular membranes, leading to blockage of voltage-gated Ca^2+^ channels and impairing calcium-dependent functions [139]. For instance, high concentrations of La^3+^ can form a stable coordination bond with calmodulin (CaM), a calcium-binding messenger protein. This interaction distorts CaM’s structure and inhibits receptor-mediated Ca^2+^ influx in root cells, compromising cellular signaling [140]. Similar effects were observed with Ca^2+^ channel inhibitors such as verapamil and ruthenium red, which, like La^3+^, reduce cytosolic calcium levels and inhibit uptake [141]. These findings suggest that La^3+^ enters root cells through both membrane and intracellular Ca^2+^ channels, affecting signal transduction via CaM-dependent protein kinases and revealing potential hormetic effects of La^3+^ at controlled levels.

Studies also show that europium (Eu^3+^) mimics Ca^2+^ in *Amaranthus caudatus*, acting within the Ca^2+^/CaM-dependent phytochrome signaling system to promote growth by facilitating calcium transport [142]. Similarly, cerium (Ce^3+^) competes with Ca^2+^ and modulates its uptake, transport, and signaling under both normal and stress conditions [98,143]. At elevated concentrations (20–80 mg/L), Ce^3+^ induces ROS accumulation, raises cytosolic Ca^2+^, activates caspase-1-like enzymes, and triggers programmed cell death in sweet potato root cells [144]. Lanthanum has been referred to as “super calcium” due to its high affinity for biomacromolecules, often replacing Ca^2+^ and interfering with calcium-regulated processes [145]. As a result, La^3+^ is classified as a Ca^2+^ channel blocker, capable of adhering to calcium-binding sites on the cell membrane and inhibiting Ca^2+^ uptake and distribution [146]. According to Kovariková et al. [147], La^3+^ may even outcompete Ca^2+^ for binding at Ca^2^-specific sites. A strong negative correlation between La and Ca concentrations in citrus leaves further supports this antagonism [92]. In addition, La^3+^ can disrupt potassium channels, reduce ATP synthesis, and impair calcium regulation, collectively compromising cell viability [148].

Ca^2+^ signaling components, such as calmodulin (CaM), play vital roles by activating enzymes, including protein kinases and phosphatases [149]. At low concentrations, La^3+^ may preserve the structural integrity and function of CaM, but at high levels, it destabilizes CaM, inhibiting its role in signal transduction [106]. Likewise, Eu^3+^ may substitute for Ca^2+^ in CaM-dependent pathways and enhance plant development by supporting calcium transport across the plasma membrane [145]. Thus, LREEs strongly influence calcium signaling pathways, affecting plant development and stress responses. The detailed mechanism of LREE-mediated calcium signaling is illustrated in Figure 3.

### 9.2. LREEs Regulate Antioxidant-Mediated Defense Response

LREEs have been reported to play crucial roles in plant stress tolerance by modulating antioxidative defense mechanisms. Plants’ antioxidant defense systems are regulated by LREEs in a concentration-dependent manner. At low concentrations, LREEs enhance ROS scavenging and improve stress tolerance by increasing the activity of key antioxidant enzymes such as superoxide dismutase (SOD), catalase (CAT), and peroxidases (POD), thereby maintaining redox homeostasis [76,147]. Antioxidant activation generally predominates below tissue concentrations of approximately 50 mg kg^−1^, whereas excessive accumulation leads to overproduction of ROS, lipid peroxidation, and membrane damage. LREEs readily enter cells and organelles, interact with biomolecules, and modulate both enzymatic and non-enzymatic antioxidant systems. For instance, La improved antioxidant enzyme activity in rice and maize [32] and increased laccase activity in *Shiraia bambusicola* [150], although excessive application (>200 µM) suppressed esterase activity [151]. La has also been shown to reduce ROS generation and lipid peroxidation in rice seeds [152], mitigate salinity stress in tomato seedlings through activation of antioxidant enzymes and reduction of MDA and H_2_O_2_ [153], and enhance P-use efficiency in *Vigna angularis* under P deficiency [110]. Furthermore, La promoted Cu uptake in Cu-stressed rice [123] and restricted Cd uptake in wheat [114]. Similarly, Ce improved drought and salt tolerance by enhancing antioxidative capacity, maintaining Na^+^/K^+^ homeostasis, and supporting osmotic adjustment [111,154], while Ce^3+^ reduced ROS and stabilized membranes in spinach chloroplasts [155]. Other LREEs such as Nd and Pr also contributed to stress mitigation by improving the ascorbate–glutathione cycle under Cr stress in wheat and Cd stress in rice, respectively [116,117]. Notably, cerium oxide nanoparticles have emerged as promising modulators of plant stress tolerance, improving salt tolerance and root development in cotton and cucumber [156,157] and stimulating Arabidopsis lateral root development via ROS and Ca^2+^ signaling [128].

In addition to antioxidant regulation, LREEs influence osmolyte adjustment, which is critical for plant survival under salinity, drought, and temperature fluctuations. Elements such as La, Ce, and Sc promote the accumulation of compatible solutes, including proline, glycine betaine, and soluble sugars, thereby enhancing osmotic balance and stress tolerance [158,159]. However, excessive concentrations may disrupt osmolyte metabolism and impair osmoregulation, leading to growth inhibition and reduced stress resilience [160]. Thus, while LREEs offer significant potential in modulating both antioxidative and osmolyte-based defense pathways, careful regulation of their concentrations is essential to harness their benefits while avoiding phytotoxic effects in agricultural systems.

### 9.3. LREEs and Secondary Metabolism

LREEs have a major impact on secondary metabolism (SMs) in plants, which includes the formation of alkaloids, flavonoids, and phenolics [161]. These substances are essential for plant defense and stress tolerance. According to recent research, exposure to LREE may increase the accumulation of bioactive molecules under mild stress by upregulating important enzymes in secondary metabolic pathways [162]. High amounts, however, may decrease metabolite production and interfere with metabolic fluxes. Enhanced accumulation of phenolics and flavonoids under low La exposure associates with increased activity and transcript abundance of phenylalanine ammonia lyase and chalcone synthase, although upstream regulatory factors remain unresolved. Like heavy metals (HMs), LREEs can inhibit enzyme activities at toxic concentrations; however, at lower levels, they enhance antioxidative enzymes and secondary metabolite production. For example, LREEs positively affected the activities of CAT, POD, and SOD, as well as the accumulation of carotenoids and phenolic compounds in cucumber and rice [163]. La^3+^ reduced ROS production and membrane lipid peroxidation in rice and groundnut seeds while enhancing POD, SOD, amylase activity, nodulation, coleoptile formation, biomass production, and nitrogenase activity [163]. Enzymes regulating SM biosynthetic pathways appear to respond to LREE availability, thereby stimulating metabolite production. In *Crocus sativus*, for instance, Ce^3+^ and La^3+^ synergistically induced carotenoid crocin accumulation when added to the growth medium [164]. In *Salvia miltiorrhiza*, application of La, Ce, and Pr at concentrations of 50–300 µM showed a concentration-dependent effect, where high REE levels reduced SM production, while flavonoids, total phenols, and tanshinones accumulated more strongly at concentrations above 100 µM [165]. Similarly, Ce promoted ginsenoside biosynthesis up to 2.7-fold in hairy roots of *Panax ginseng*, likely mediated via JA signaling and defense pathways [166]. Pr also altered flavonoid content in *Scutellaria viscidula* roots by modulating POD, polyphenol oxidase (PPO), and phenylalanine ammonia-lyase (PAL) activity [165], while Ce enhanced both resilience and the medicinal flavonoid and polyphenol content of *Dendrobium nobile* [167]. Beyond these effects, Batista-Oliveira et al. [168] reported that Gd protects *Arabidopsis thaliana* from *Botrytis cinerea* infection by activating JA- and ethylene-dependent defense responses.

### 9.4. Transcriptional Regulation of LREE

The “transcriptional regulation” of LREEs refers to the modulation of gene expression in response to their concentrations in the plant environment. Zheng et al. [94] identified NRAMP REE Transporter 1 (NREET1), a REE transporter from the hyperaccumulating fern *Dicranopteris linearis*, which mediates uptake and transfer of REEs from the cell wall into the cytoplasm, with greater efficiency for light REEs compared to heavy REEs. Evidence from Arabidopsis expression studies further supports the role of NREET1 in REE transport. In rice seedlings, Sun et al. [122] demonstrated hormetic effects of La and Gd on the cell cycle of root tips, where low doses facilitated G2/M phase progression, while higher doses inhibited G1/S phase transition. Similarly, He et al. [169] showed that La (III) induced dynamic changes in DNA methylation of Arabidopsis roots, initially activating CMT3, DRM2, and DNMT2, followed by later-stage activation of MET1, CMT1, CMT2, CMT3 (methylases), and ROS1, DME, DML2, DML5a, and DML5b (demethylases). These modifications highlight the regulatory influence of LREEs on epigenetic processes. Moreover, Jiao et al. [170] reported that La (III) altered the expression of stomatal opening-related genes, including OST1, OST2, PHYB, FT, CO, and CRY2, at both low (≤30 μM) and high (≤80 μM) concentrations. Together, these studies suggest that LREEs regulate transcriptional networks underlying plant growth, development, and defense responses, often in a concentration-dependent manner. Current transcriptional evidence remains limited to a small number of candidate transporters and epigenetic responses. Functional validation using loss-of-function mutants and comparative expression profiling across species is required before regulatory networks can be deciphered.

## 10. LREEs and Plant Growth Regulators Crosstalk

Plant hormones are central regulators of growth and stress responses, and their interactions with LREEs represent a key mechanism in plant development. REEs influence auxin dynamics by modulating tryptophan biosynthesis and reducing IAA catabolism; for example, La increased IAA levels in cucumber and rice [162] and reversed ABA-inhibited seed germination in Arabidopsis while promoting root hair development [171]. In rice, La^3+^ induced *OsGH3.4* expression, suppressing IAA-related expansins under Cu stress [123], while in Avena coleoptiles, La enhanced auxin interactions by altering plasma membrane lipid–protein complexes [172]. Nd has also been shown to increase endogenous IAA and auxin: cytokinin ratios, thereby accelerating root primordium formation [173].

Beyond auxins, REEs interact with other PGRs in diverse ways. La and Nd enhanced GA3-induced α-amylase activity in the aleurone layer of wheat, thereby improving nutrient mobilization [174,175]. Lanthanum suppressed ABA activity during stomatal closure [176] and cytokinin activity during bud initiation [177], while nitric oxide (NO) antagonized La activity by restricting its uptake in rice [178]. REEs also affect ethylene- and SA-related signaling; for instance, La-induced systemic endocytosis required AtrbohD- and JA-mediated systemic signaling [95], Ce interacted positively with SA to regulate growth and nutrient uptake [179], and Gd triggered JA- and ET-dependent defense responses against *Botrytis cinerea* [168]. At the physiological level, LaCl_3_ treatments increased IAA, ZR, and GA levels while decreasing ABA levels, thereby improving photosynthesis and grain filling in maize [32]. However, GA and Ce interacted negatively under CeNP stress in wheat, reducing growth and antioxidant performance [180], and Ce combined with ABA altered alfalfa germination and antioxidant activity under stress [181]. Collectively, these findings demonstrate that LREEs interact extensively with auxins, gibberellins, ABA, cytokinins, SA, JA, and ET to regulate plant growth, development, and stress responses (Table 5). Available data support indirect modulation of auxin transport and sensitivity by LREEs, while evidence for direct interactions with hormone receptors or biosynthetic enzymes remains insufficient.

## 11. Conclusions and Future Perspectives

Efficient plant nutrient management is crucial for food, the environment, and global nutritional sustainability. The roles of macro, micro, and beneficial elements in agricultural production have been widely studied, and their uptake, transport, bioavailability, crosstalk, and functions in plants are being investigated at the molecular level. The mineral nutrients, such as LREEs, have also been shown to be effective at appropriate concentrations, but their in-depth study remains limited. Thus, understanding the mechanisms of uptake and transport is critical for elucidating their specific functions. In soil, LREEs often compete with other essential minerals for their uptake; however, specific uptake mechanisms remain to be elucidated. Moreover, crosstalk between LREEs and other essential nutrients and PGRs is equally important for improving plant nutrient use efficiency and signal transduction mechanisms. LREEs are effective against ROS and the production of secondary metabolites; their role in abiotic stress tolerance is poorly exploited. Most of the research on these elements is done on lanthanum and cerium due to their soil abundance; therefore, there is an opportunity to explore other elements for their effects on plant growth and development. In emerging research areas, LREEs have been used as elicitors for secondary metabolite production. Also, the limited application proved beneficial for plant growth and development, underscoring the need for further research in this direction for crop applications. Future research should prioritize field-scale dose–response studies, uptake and transporter specificity, long-term soil accumulation risks, and crop-specific safety thresholds. Without these data, the agricultural application of light rare earth elements remains scientifically premature. Also, the role clarity (Growth stimulation at lower concentration/growth inhibition at higher concentrations) of LREEs needs more studies to decipher molecular mechanisms. Another priority area should be the molecular and biochemical evidence of LREEs as replacements for Ca^2+^ signaling and targets. Similarly, LREE interactions with phytohormones and the plant microbiome should be explored in future studies. This review compiled in-depth information on LREEs and their impact on plant growth and development, thus opening a new avenue for researchers to consider using LREEs in sustainable ways for sustainable crop production and to ensure food security in the future.

## Figures and Tables

**Figure 1:**
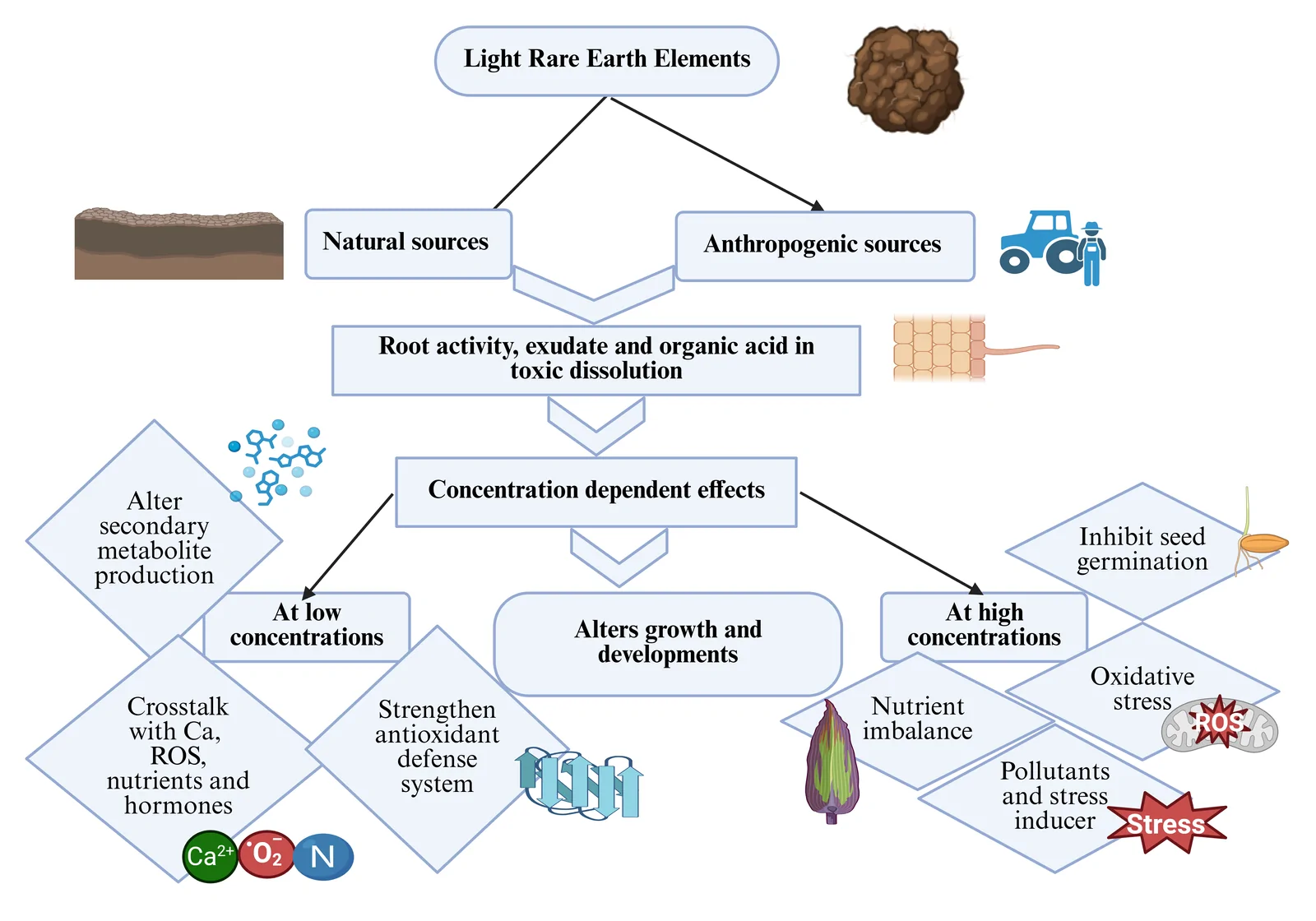
Schematic presentation of the effects of light rare earth elements (LREEs) on plant growth and development.

**Figure 2:**
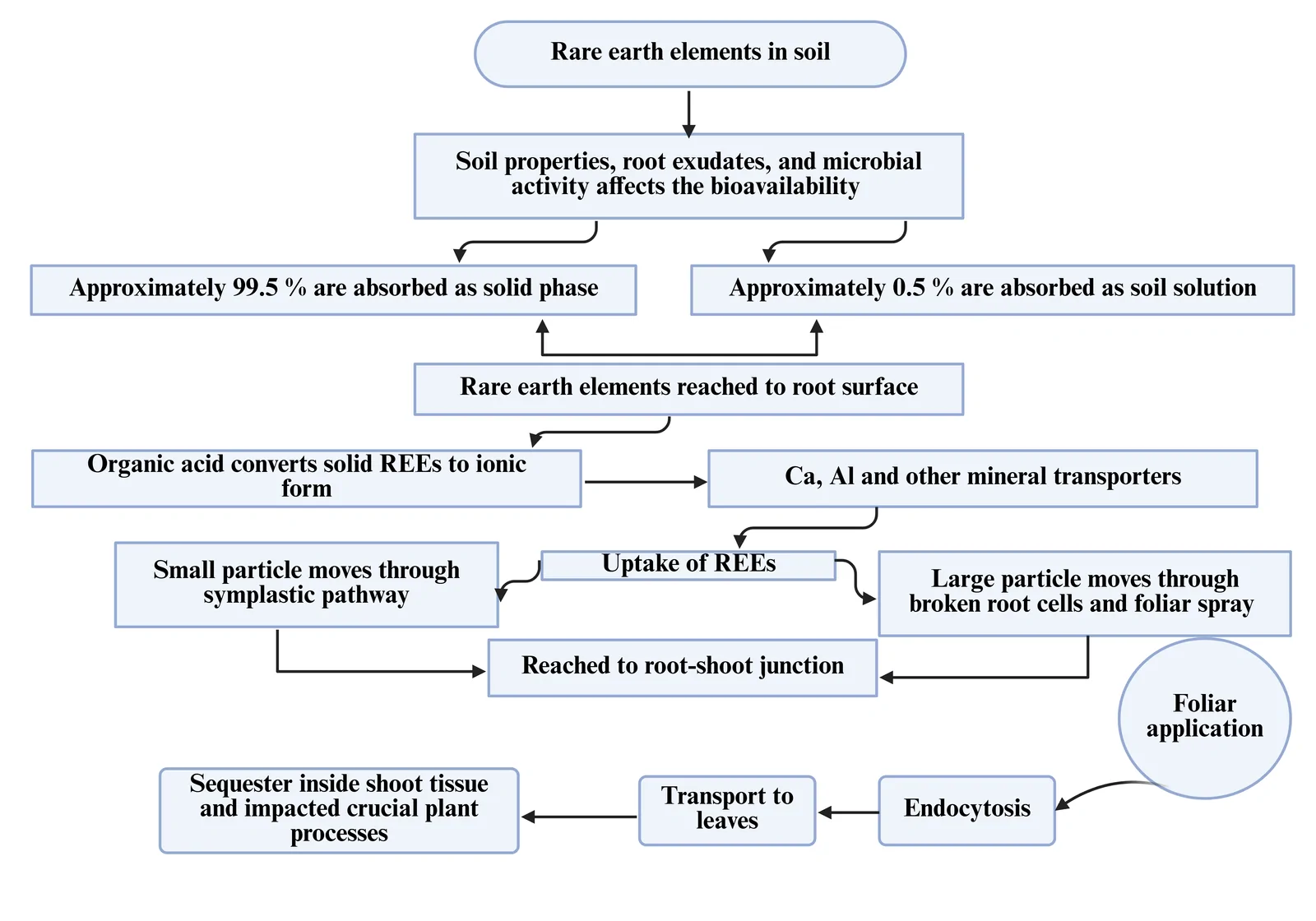
Highlights the general uptake and transport mechanisms of light rare earth elements from soil to plant.

**Figure 3:**
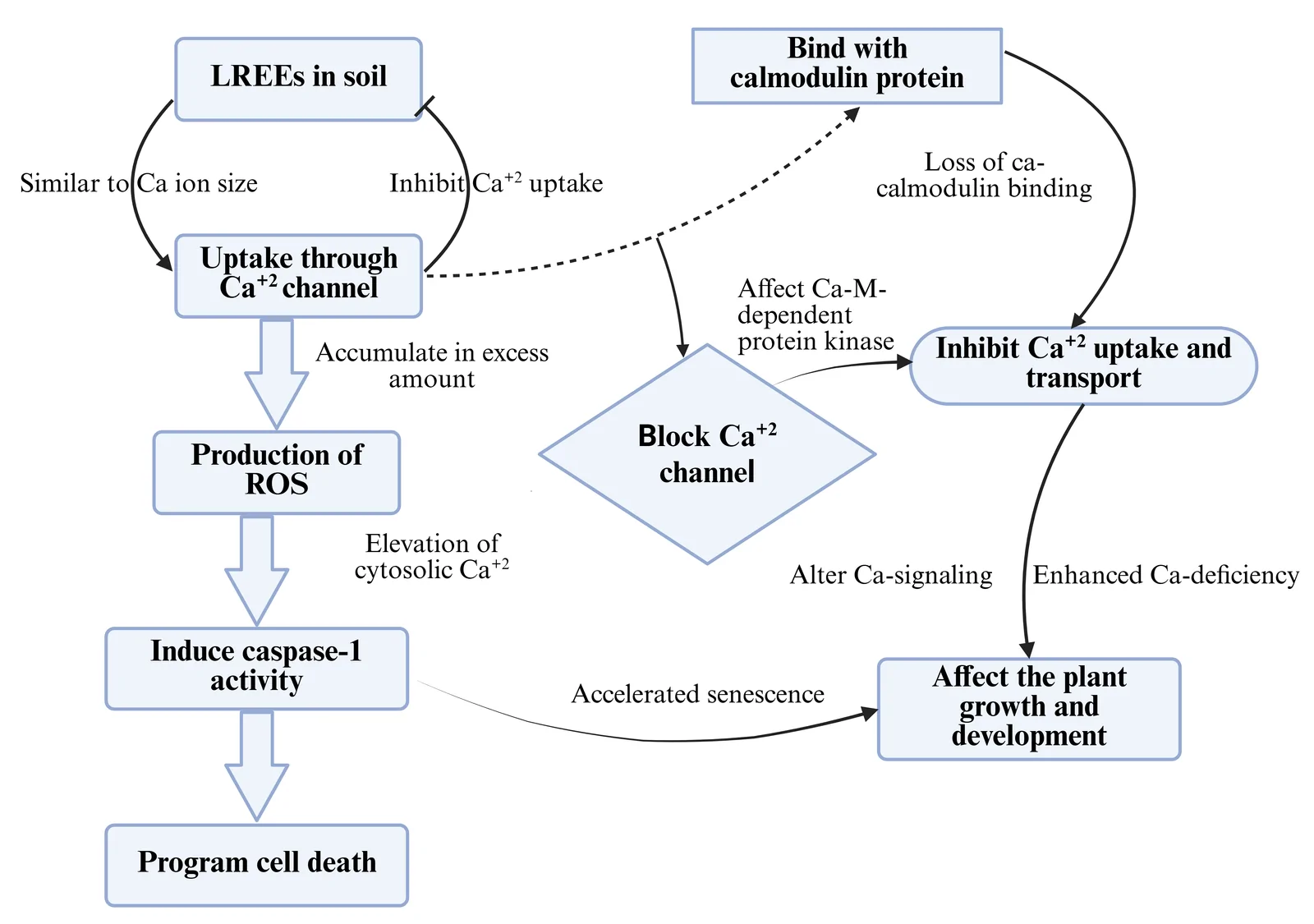
Ca signaling mechanisms under light rare earth elements (LREEs) and their effects on the plant’s functional processes.

**Table 1:** Sources and concentrations of LREE in the natural environment.

Element	Natural Source	Concentration mg/kg [min ^a^–max ^a,b^ (Mean ^c^)]	Air Concentration ^d^ (pg/m^3^)
Lanthanum (La)	Earth crust * (Bastnaesite, Monazite)	5–39 (35)	South Pole 0.2–1.4; Spitsbergen 5–30; United States 500–9000
Cerium (Ce)	Earth’s crust * (Bastnaesite, Monazite, Loparite)	20–70 (66)	South Pole 0.8–5; Spitsbergen 20–60; Great Britain 330–550 (ng/m^3^)
Praseodymium (Pr)	Earth’s crust *	3.5–9.2 (9.1)	60
Neodymium (Nd)	Earth’s crust *	12–41.5 (40)	~300
Promethium (Pm)	Radioactive & artificially created	NA	NA
Samarium (Sm)	Earth’s crust *	4.5–8 (7.0)	South Pole 0.03–0.1; Great Britain 20–40; United States 70–1000
Europium (Eu)	Earth’s crust *	0.14–2 (2.1)	South Pole 0.004–0.02; Spitsbergen 1–2; Germany 5–80

^a^ [43]; ^b^ [44]; ^c^ [45]; ^d^ [46]. * Mostly in the deposits of bastnaesites, monazites, or xenotimes for extraction [47].

**Table 2:** Anthropogenic sources of rare earth elements (REEs) and their ecological effects and environmental significance.

Anthropogenic Source	LREEs *	Environmental Significance	Reference
NiMH batteries	Ce, La, Nd	REEs are released into soil and water systems through disposal and recycling	[53,54]
Permanent magnets/Voice coil motors	Nd, Pr	E-waste and industrial processing both contribute to environmental accumulation	[55]
Fluorescent lamp phosphors	Ce, La	Leaching into aquatic systems and landfills from abandoned lamps	[56]
Fe–Nd–B magnets	Nd	Electronic trash and green technology are major sources of REE	[57]
Sm–Co magnets	Sm	Localized pollution is a result of high-tech applications and industrial waste	[58]
TV tubes and computer monitors	Eu, Y	REEs are being released into the environment via legacy electronic garbage	[59]
Coal and coal by-products, iron ore tailings, apatite, phosphate by-products	REEs	The amount of REEs in soils and agroecosystems is increased by mining and fertilizer use	[11,60]
Mine waste and industrial landfill by-products (phosphogypsum, red mud, slags, coal ash, wastewater)	REEs	Leaching, irrigation, and atmospheric deposition are the main ways by which REEs get contaminated	[13,61]
Agricultural inputs (fertilizers, wastewater irrigation)	La, Ce, Pr, Nd	Improve plant uptake and soil buildup via the soil–plant continuity	[11,19]
Atmospheric deposition and airborne particles	LREEs	Plant accumulation is facilitated by the foliar uptake route	[19]
Aquatic contamination (industrial discharge)	Ce, Nd, Gd, Dy, Yb, Ho, Lu	Affects the health of ecosystems and aquatic plants like *Lemna minor*	[62]
Controlled application in agriculture	La	Governs stomatal function, N–C metabolism, and yields under ideal doses	[63]

* Lanthanum (La), cerium (Ce), praseodymium (Pr), neodymium (Nd), samarium (Sm), europium (Eu), yttrium (Y), gadolinium (Gd), dysprosium (Dy), ytterbium (Yb), holmium (Ho), and lutetium (Lu).

**Table 3:** Hyperaccumulation of rare earth elements (REEs) in Plant Species: Concentrations, Uptake Mechanisms, and Physiological Insights.

Hyperaccumulator ^a^ Plant	Family	Element *	Max. Conc. in Leaves (mg/kg)	Mechanism	Reference
*Dicranopteris dichotoma*, *D. linearis*	Gleicheniaceae	La, Ce	1040 ± 520; 7000 ^c^	Compartmentalization; co-localization with Mn in fronds	[72,73,74]
*Phytolacca americana*, *P. icosandra*	Phytolaccaceae	La, Y	1012; 13,000 ^c^	Complexation with amino acids (asparagine, histidine, citrate) enabling xylem transport	[17,75]
*P. americana*, *P. acinosa*, *P. bogotensis*, *P. clavigera*	Phytolaccaceae	REEs ^b^	10,000; 8000; 4000; 1000	Preferential HREE transport, Mn antagonism, anthocyanin induction, vacuolar sequestration	[76,77]
*Dicranopteris strigosa*	Gleicheniaceae	La, Ce, Nd, Pr	up to 3358–7000 ^c^	Histidine-mediated desorption and enhanced uptake	[17,78,79]
*Zygophyllum album* (*Tetraena alba*)	Zygophyllaceae	La	11.05	High translocation factor (TF ~6), efficient phytoextraction	[80,81]
*Blechnum orientale*, *B. niponicum*	Blechnaceae	REEs ^b^	1022 ^c^; 115	Likely vacuolar storage and cell wall binding	[77,79,82]
*Carya* spp. (multiple species)	Juglandaceae	REEs ^b^	166–2300	Positive correlation with Ca, Mg, Mn; ploidy-dependent accumulation	[13,83,84,85]
*Pronephrium simplex*, *P. triphyllum*	Thelypteridaceae	REEs ^b^	1234; 1027	Peptide binding enhances LREE accumulation	[17,86]
*Athyrium* spp.	Athyriaceae	REEs ^b^	187–270	Likely passive accumulation; limited mechanistic data	[77,87]
*Stenoloma chusanum*	Lindsaeaceae	REEs ^b^	725	Unknown (likely apoplastic binding)	[79]
*Woodwardia japonica*	Blechnaceae	REEs ^b^	367	Unknown	[79]
*Adiantum* spp., *Gleichenia* spp.	Adiantaceae	REEs ^b^	up to 3340 ^c^	Hyperaccumulation via cell wall and vacuolar sequestration	[77,78]
*Asplenium* spp.	Aspleniaceae	REEs ^b^	12–40	Limited accumulation; possible exclusion strategy	[78]
*Dryopteris erythrosora*	Dryopteridaceae	REEs ^b^	175	Mn–REE co-regulation suggests shared pathways	[77,82]
*Onoclea sensibilis*	Onocleaceae	La, Ce, Sm, Gd, Yb	~200	Multi-element accumulation pattern	[77]
*Alsophila sternbergii*	Cyatheaceae	Ce	484	Likely vacuolar sequestration	[88]
*Pteridium aquilinum*	Dennstaedtiaceae	REEs ^b^	131	Low-level accumulation	[79]

* Lanthanum (La), cerium (Ce), praseodymium (Pr), neodymium (Nd), samarium (Sm), europium (Eu), ytterbium (Yb), and yttrium (Y). ^a^ Plants that contain in their dry weight foliar tissue > 1000 μg g^−1^, when growing in their natural habitat [70]. ^b^ REE hyperaccumulator plants exceed 1000 µg g^−1^ of total REE in the frond or have a bioconcentration factor (BCF = C_foliage_/C_substrate_) greater than 1 [89]. ^c^ Eight elements (La, Ce, Nd, Sm, Eu, Tb, Yb, Lu).

**Table 4:** Concentration-dependent physiological and biochemical effects of light rare earth elements (LREEs) on plant growth, development, and stress responses.

Process	Study Model	LREE(s) Used	Concentration	Observed Effect	Updated References
Accumulation in tissues	General species	Mixed LREEs	Low vs. High	Roots accumulate more LREEs; high levels induce compartmentalization and toxicity	[17,80,119,120]
Seed germination	Salvia	La^3+^	20 vs. 100 mg/kg	Low dose enhances germination; high dose inhibits growth	[121,122]
Germination under stress	Rice	La-chelate	20 mg/L	Improves germination and root traits under metal stress	[71,123]
Growth & chloroplast ultrastructure under stress	Soybean	La^3+^	Low vs. high under acid rain	Low improves growth; high causes chloroplast damage	[76,124]
Seed imbibition	Tomato	CeCl_3_	–	Increased membrane permeability and seed hydration	[121,125]
Germination rate	Wheat, Rice	Nd_2_O_3_	0.5 vs. 5 mg/L	Low improves germination; higher enhances biomass	[71,126]
Seedling growth	Sweet pepper	Ce, Nd, Pr	0.5–1.5 ppm	Improved vigor and early growth	[76,127]
Photosynthesis efficiency	Rice	Ce^3+^, La^3+^	0.2–4.2 μM	Enhanced chlorophyll, Rubisco activity, CO_2_ fixation	[106,121]
Calcium signaling	Rice roots	La^3+^	0.2–2.0 mmol L^−1^	Acts via Ca^2+^ channels and calmodulin signaling	[76,91,128]
Antioxidant metabolism	Rice	Ce^3+^	0–1.5 mM	Low promotes antioxidants; high induces ROS damage	[62,71,91]
Growth inhibition & metal interaction	Rice	Mixed REEs	0.5–1 mg/L	Growth inhibition; Fe plaque provides partial protection	[13,129]
Cell cycle regulation	Root tips	La^3+^	0.1–10 mg/L	Low promotes growth; high causes DNA damage	[71,130]
Root & mitochondrial activity	Bahiagrass	La	–	Enhanced root growth and energy metabolism	[76,111]
Cytotoxicity & genotoxicity	Onion	La, Ce	0–200 mg/L	Chromosomal aberrations and mitotic inhibition	[71,131]
Chlorophyll substitution	Switchgrass	La	–	Formation of La–chlorophyll complexes	[71,121]
Seed germination barrier	Switchgrass	La^3+^	–	Restricted penetration due to seed coat barriers	[19,132]
Photosynthesis under metal stress	Spinach	Ce^3+^	Pb^2+^ + Ce^3+^	Ce alleviates Pb toxicity and restores PSII	[76]
PSII enhancement	Spinach	La	–	La substitutes Ca in the oxygen-evolving complex	[121]
PSII photoprotection & ROS control	*Pseudostellaria heterophylla*	La^3+^, Ce^3+^	Field	Improves PSII efficiency and antioxidant defense	[98,133]
Yield & productivity	Wheat, Spinach	Nd, Ce	Adequate	Enhanced photosynthesis and biomass	[134,135]
Drought stress alleviation	Common bean	Ce^3+^	μmol range	Improved WUE, chlorophyll, survival	[76,136]

**Table 5:** Crosstalk between plant growth regulators and light rare earth elements (LREEs) in plants.

Plant Growth Regulator	Rare Earth Element	Reported Crosstalk Mechanism	References
Auxin (IAA)	Lanthanum (La^3+^)	Increases auxin accumulation and root elongation by decreasing IAA oxidase activity and increasing IAA production through tryptophan pathway activation	[17,76,111]
	Neodymium (Nd^3+^)	Enhances endogenous IAA levels and activates auxin-responsive genes to encourage adventitious root growth	[72,173]
Cytokinin	Lanthanum (La^3+^)	Antagonistic relationship with cytokinin signaling that modifies cytokinin receptor sensitivity to prevent cell division and bud initiation	[76,177]
Gibberellic Acid (GA)	Cerium (Ce, CeO_2_ NPs)	Under nanoparticle stress, negative crosstalk modifies GA signaling, resulting in changes to biomass allocation, growth, and oxidative balance	[121,180]
	Neodymium (Nd^3+^)	Enhances GA-induced α-amylase activity, reduces the GA_3_ response’s lag phase, and encourages seed germination and enzyme activation	[17,174,175]
Abscisic Acid (ABA)	Lanthanum (La^3+^)	Works in opposition to ABA in the control of stomata; also modifies Ca^2+^ signaling pathways that impact stomatal closure and prevents ABA-induced H_2_O_2_ generation	[63,171,176]
Ethylene (ET)	Lanthanum (La^3+^)	Reduced senescence and stress-induced ethylene signaling; lowers ethylene synthesis under abiotic stress (e.g., UV-B)	[76]
Nitric Oxide (NO)	Lanthanum (La^3+^)	Regulates La uptake and translocation, while antagonistic interaction governs stress reactions and ROS signaling	[71,178]
Jasmonic Acid (JA)	Cerium (Ce^3+^)	Increases the production of defense genes and JA-mediated stress signaling under abiotic stress conditions	[72,76]
Salicylic Acid (SA)	Lanthanum (La^3+^)	Regulates SA-dependent defense mechanisms and systemic acquired resistance (SAR) via ROS signaling	[71,80]

## Data Availability

No new data were created or analyzed in this study. Data sharing is not applicable to this article.
